# Rapid and non-destructive identification of *Anopheles gambiae* and *Anopheles arabiensis* mosquito species using Raman spectroscopy via machine learning classification models

**DOI:** 10.1186/s12936-023-04777-y

**Published:** 2023-11-08

**Authors:** Dickson L. Omucheni, Kenneth A. Kaduki, Wolfgang R. Mukabana

**Affiliations:** 1 Department of Physics, University of Nairobi, Nairobi, Kenya; 2 Department of Biology, University of Nairobi, Nairobi, Kenya; 3 Science for Health Society, Nairobi, Kenya

**Keywords:** Raman spectroscopy, Machine learning, Discriminant Analysis, Support Vector Machine, Logistic Regression, Melanin, Mosquito, Mosquito identification, *Anopheles gambiae*, *Anopheles arabiensis*

## Abstract

**Background:**

Identification of malaria vectors is an important exercise that can result in the deployment of targeted control measures and monitoring the susceptibility of the vectors to control strategies. Although known to possess distinct biting behaviours and habitats, the African malaria vectors *Anopheles gambiae* and *Anopheles arabiensis* are morphologically indistinguishable and are known to be discriminated by molecular techniques. In this paper, Raman spectroscopy is proposed to complement the tedious and time-consuming Polymerase Chain Reaction (PCR) method for the rapid screening of mosquito identity.

**Methods:**

A dispersive Raman microscope was used to record spectra from the legs (femurs and tibiae) of fresh anaesthetized laboratory-bred mosquitoes. The scattered Raman intensity signal peaks observed were predominantly centered at approximately 1400 cm^−1^, 1590 cm^−1^, and 2067 cm^−1^. These peaks, which are characteristic signatures of melanin pigment found in the insect cuticle, were important in the discrimination of the two mosquito species. Principal Component Analysis (PCA) was used for dimension reduction. Four classification models were built using the following techniques: Linear Discriminant Analysis (LDA), Logistic Regression (LR), Quadratic Discriminant Analysis (QDA), and Quadratic Support Vector Machine (QSVM).

**Results:**

PCA extracted twenty-one features accounting for 95% of the variation in the data. Using the twenty-one principal components, LDA, LR, QDA, and QSVM discriminated and classified the two cryptic species with 86%, 85%, 89%, and 93% accuracy, respectively on cross-validation and 79%, 82%, 81% and 93% respectively on the test data set.

**Conclusion:**

Raman spectroscopy in combination with machine learning tools is an effective, rapid and non-destructive method for discriminating and classifying two cryptic mosquito species, *Anopheles gambiae* and *Anopheles arabiensis* belonging to the *Anopheles gambiae* complex.

## Background

Malaria remains a serious threat to human life worldwide. It is a serious cause of morbidity and mortality, with the majority of cases and deaths occurring in sub-Saharan Africa. According to statistics from the World Health Organization (WHO), it is estimated that 247 million cases and 619,000 deaths occurred worldwide due to the disease in the year 2021 [1]. The highest burden of the disease was bone in sub-Saharan Africa, which accounted for 95% of cases and 96% of deaths.

Malaria is transmitted from one human to another primarily via the bites of female *Anopheles* mosquitoes. To this end, female mosquitoes of the genus *Anopheles* must contract infection with *Plasmodium* parasites by feeding on blood from an infected person, supporting the sexual cycle of *Plasmodium* parasites, and subsequently delivering the infective sporozoites to a susceptible human host during the next blood-feeding session [2]. Based on this knowledge, malaria prevention by reducing human-vector contact is paramount and has been the main preventive strategy hitherto. This is usually deployed in the form of indoor residual spraying (IRS), insecticide-treated bed nets (ITNs), larval control, and outdoor residual spraying [3].

The *Anopheles gambiae* complex includes some of the most efficient malaria vectors worldwide. It is known to be composed of at least seven morphologically indistinguishable species, namely, *Anopheles gambiae* (i.e. the nominal taxon), *Anopheles arabiensis*, *Anopheles bwambae*, *Anopheles melas*, *Anopheles merus*, *Anopheles quadriannulatus*, *Anopheles coluzzii*, and *Anopheles amharicus* [4]. The first two species are the most widely distributed in Africa [5]. Identifying *An. gambiae* and *An. arabiensis* is critical in malaria control programmes because these two species have distinct behaviours and habitats and require different approaches to control. *Anopheles gambiae* is generally the predominant species in environments with high humidity and rainfall, whereas *An. arabiensis* is more common in regions with low rainfall [5, 6]. Both species often occur in sympatry across a wide range of tropical Africa [5, 7].

Identifying the species responsible for malaria transmission in a given area allows the application of targeted interventions that are more effective and efficient than the use of broad-based control measures. *Anopheles gambiae* is an endophilic mosquito species [8, 9], meaning that it rests indoors after blood feeding. It is primarily a nighttime feeder and is highly anthropophilic [9], meaning that it feeds almost exclusively on humans. Owing to its feeding and resting habits, *Anopheles gambiae* is primarily controlled using indoor residual spraying (IRS) and insecticide-treated bed nets (ITNs). In contrast, *Anopheles arabiensis* is an exophilic mosquito, meaning that it rests outside after feeding. It is opportunistic in its feeding habits, feeding on both humans and animals [10]. Because it rests outdoors, IRS may not be as effective against *An. arabiensis*. Instead, other measures such as larval source management and outdoor residual spraying may be necessary to control this species.

Distinguishing between the two cryptic species is also important for monitoring insecticide resistance [11]. The widespread use of insecticides for malaria control has led to the development of resistance in mosquito vector populations. Monitoring insecticide resistance is critical to ensure that control measures remain effective. Additionally, correct identification can aid in tracking changes in malaria transmission patterns [12]. Monitoring changes in mosquito abundance and species composition can provide valuable information regarding malaria transmission dynamics. This information can be used to adjust control strategies and prevent malarial epidemics.

The need to identify these two species is an age-old problem. Their cryptic nature has prompted scientists to develop identification techniques that extend beyond visible morphological features. In most cases, these techniques are molecular techniques. Allozyme electrophoresis [13] and analysis of polytene chromosomes [14] may be considered the oldest techniques for identifying mosquitoes in the *An. gambiae* complex. In the former method, starch gel electrophoresis of allozymes was used to identify the members of the *An. gambiae* complex. Gel electrophoresis is a method used to separate macromolecules, mainly proteins, based on their size and charge. Macromolecules are embedded in a porous gel, such as starch, and an electric field is applied across the gel. The migration of molecules in the porous gel under the influence of an electric field separates the molecules by size. The process is tedious and time-consuming, as it requires manual identification of protein products at particular loci. In the latter method, the evolution of the *An. gambiae* complex was interpreted from the polytene chromosome banding sequences. Polytene chromosomes are giant chromosomes that are commonly found in dipteran flies. Although successful, this method is limited to half-gravid female mosquitoes [15].

Cuticular hydrocarbon analysis using gas chromatography has also been explored for the identification of *An. gambiae* complex species. Gas chromatography can be used to separate volatile compounds without causing decomposition. This enabled the determination of the relative abundances of the present compounds. Carlson & Service [15] observed that relative abundances between the following compounds could be used to distinguish the species: n-hexacosane and n-heptacosane, 13-methyl hentriacontane and n-hentriacontane, and dimethyl nanotria-contane and dimethyl hententra-contane. However, the instrumentation of gas chromatography limits its use in laboratory environments and not in field applications.

Most techniques for identifying the sibling species of *An. gambiae* complex, as observed from recent literature, are focused on the analysis of DNA [16–24]. DNA is a biological molecule that encodes the genetic information of an organism. Currently, DNA analysis is performed using Polymerase Chain reaction (PCR) amplification. PCR is used to select specific portions of the genome (a sequence of bases) of an organism to be replicated several times to a reasonable quantity for analysis. PCR techniques have high accuracy, specificity, and sensitivity. Currently, they are the gold standard for identifying species in the *An. gambiae* complex. However, PCR is a time-consuming, labour-intensive, and expensive process that requires specialized laboratory conditions and highly skilled personnel to obtain good results.

Matrix-assisted laser desorption/ionization time-of-flight mass spectrometry (MALDI-TOF-MS) has been explored for discriminating sibling species within the *An. gambiae* complex. MALDI-TOF-MS uses laser energy and an absorbing matrix to ionize large molecules such as proteins while minimizing fragmentation. Using MALDI-TOF-MS, mosquito leg protein extracts have been found to be adequate for identifying mosquitoes at the species level [25–27]. Similarly, optical spectroscopy techniques, such as near-infrared (NIR) and mid-Infra-red (MIR) spectroscopy, have been investigated in insect taxonomic and age grading studies. These include the identification of species of beetles [28], *Drosophila* species [29], cryptic *Tetramorium* ant species [30], and the *An. gambiae* complex [30–34]. NIR and MIR absorption and reflectance spectroscopy probes the vibrational states of molecules and provides a spectral fingerprint of the chemical compound under investigation. While cuticular lipids and hydrocarbons are the main molecules that provide essential classification information in insect taxonomic studies using NIR spectroscopy [30, 31], MIR spectroscopy has consistently revealed proteins, waxes and chitin as the overall indicators of spectra intensity [32–34]. The three techniques, MALDI-TOF-MS, MIR and NIR spectroscopy, have the advantage of being rapid compared to previously discussed methods. However, in MALDI-TOF-MS, the compound to be investigated must be extracted and embedded in a laser-absorbing matrix for analysis, which is a time-consuming process. MIR spectroscopy, on the other hand, is generally a non-destructive method. However, the recently reported attenuated total reflectance (ATR) method of spectral measurement on dried mosquito samples [32–34] can potentially be destructive as the sample is pressed against a crystal using an anvil. In addition, the process of drying the samples for more than a day may also lead to delays.

From the extensively reviewed literature, no studies have used Raman spectroscopy as a tool to distinguish these two species. Recently, the capability of Raman spectroscopy to discriminate and classify mosquito genera was demonstrated [35]. Cuticular melanin was found to be a potential biomarker for discrimination. In this work, the utility of Raman spectroscopy for discriminating and classifying two sibling species based on cuticular melanin signatures is demonstrated.

## Methods

### Mosquito rearing and preparation of samples for Raman spectroscopy

*Anopheles gambiae* and *An. arabiensis* colonies were maintained following standard rearing protocols in insectaries at the Department of Biology at the University of Nairobi. Adult mosquitoes were held in 30 × 30 × 30 cm cages in separate temperature-, light-, and humidity-controlled rooms. The rooms were kept at a temperature of 27–28 °C, humidity of 70–80% and a photoperiod of 12-h light and 12-h darkness. In each cage, the mosquitoes laid eggs in ovicups containing cones of filter paper placed in water. The eggs were transferred to trays filled with water, where they hatched into larvae. The larvae were fed TetraMin® baby fish food. The adults were fed 6% glucose solution soaked in filter paper wicks. Confirmatory PCR was performed to maintain colony integrity.

To prepare for measurements, fresh 3-day old adult mosquitoes were taken from rearing cages and anaesthetized using chloroform. This was performed by enclosing separate groups of insects in enclosed chambers that contained open bottles of chloroform for six hours. This step was not necessary for the measurement process but was performed to ensure that the mosquito samples were kept intact with all body parts.

### Raman spectroscopy measurements

**Fig. 1:**
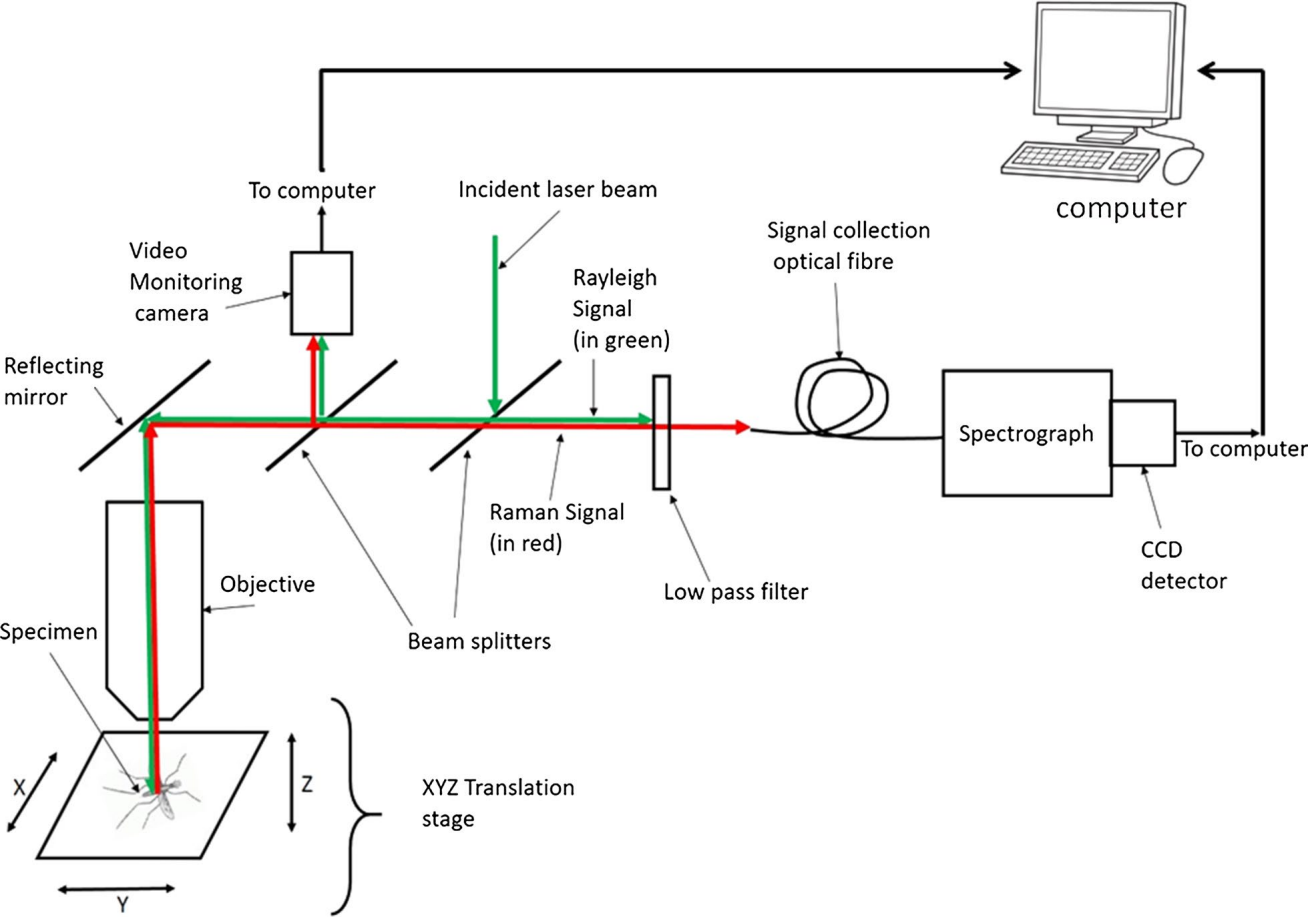
Schematic representation of the dispersive Raman microscope. The sample is placed on the X–Y stage. Red and green arrows indicate photon delivery routes

A total of 397 mosquitoes (*An. gambiae*, n = 201; *An. arabiensis*, n = 196) were used in this study. Each mosquito was measured by randomly taking five spectra of the insect’s femurs and tibiae. Each spectrum was taken using 10 s integration time with five accumulations on a Technos^®^ dispersive Raman microscopy system. The system had the following parameters: 532 nm excitation laser, 600 grooves/mm gratings, and × 10 infinity-corrected dry microscope objective with a Numerical Aperture of 0.25. The laser power was continuously adjusted via visual inspection of the sample to avoid sample burning. Figure 1 shows a schematic of the instrument setup, with the sample (insect) placed on the X–Y translation stage. Photons from the laser (indicated by green arrows pointing toward the sample) were delivered through the microscope objective to the mosquito legs (femurs and tibiae), which were placed on a Raman-grade Calcium Fluoride microscope slide (Crystran Ltd, UK, Batch No. 60373). Femurs and tibiae were chosen for their relative ease of being focused under the microscope and because they did not contain signals emanating from other conditions such as food consumed by the mosquitoes and gonotrophic status. Furthermore, mosquito legs have been successfully used to classify mosquitoes [25, 27, 35]. Rayleigh and Raman scattered photons (indicated as green and red arrows respectively pointing away from the sample) in Fig. 1 were collected by the same microscope objective with an optical low pass filter blocking the former. The Raman signal was collected via an optical fibre and spectrograph for digitization using a charge-coupled device (CCD) (Peltier cooled to − 76 °C) connected to a computer for display and storage. Wavenumber calibration was done by interpolating the laser line and the strong silicon Raman shift positioned at 520.5 ± 4 cm^−1^.

### Data pre-processing

Data processing was performed according to previously published protocols by Ryabchykov et al. [36] and Morais et al*.* [37]. Five spectra from each mosquito were averaged, and the average was considered as a single spectrum from the mosquito. Each averaged spectrum was first smoothed with a Savitzky Golay convolution digital filter of order 5 and a frame length of 21 pixels. The Savitzky Golay mask is a low-pass filter that suppresses noise signals that generally have high frequencies. This was followed by a baseline correction procedure employing the Vancouver algorithm [38] with a 5th-order polynomial to subtract the fluorescence signal and leave a clean Raman signal. Vector normalization was applied to each Raman spectrum to account for the intensity variation due to experimental factors such as changes in the sample focus. All preprocessing procedures were performed using scripts developed in the Matlab^®^ 2018 software.

### Classification modelling

Four machine-learning models were trained and tested to discriminate and classify the two mosquito species: Linear Discriminant Analysis (LDA), Quadratic Discriminant Analysis (QDA), Logistic Regression (LR), and Support Vector Machine (SVM). LDA and QDA are algorithms based on Mahalanobis distance calculations between samples of each class, which can use Bayesian probability terms to correct classes of different sizes. Unlike LDA, which assumes a pooled covariance matrix resulting in a linear boundary, QDA forms a separate covariance model for each class thus separating the classes by a quadratic boundary. Similarly, LR is a linear model, used to model binary classes based on one or more variables. In this case, the binary classes were assumed to closely fit an underlying probability distribution. The goal of the model was to estimate the true parameters of the underlying probability density function for discrimination and classification. Finally, the data were modelled using an SVM. An SVM is an inherently binary linear classifier that may allow the nonlinear transformation of data by the inclusion of kernel tricks. SVM fits a linear decision boundary between classes while seeking to maximize the margin between them, defined by the closest samples on the border, called support vectors. In this study, we used a quadratic kernel to account for nonlinearity in the classification problem. In all the four models, Principal Component Analysis PCA was used to extract features for classification. PCA is a matrix factorization algorithm that decomposes the original data matrix into three matrices as follows:

1 $$\mathbf{X}=\mathbf{T}{\mathbf{P}}^{\mathbf{T}}+\mathbf{E}$$

where **T** is a matrix containing scores, **P**^**T**^ is the transpose of the matrix containing loadings, and **E** is a residual. The score matrix **T** has its columns ordered from the first most important latent variable, with each subsequent variable’s importance reduced. This implies that the first few latent variables can be used to reduce the dimensions of the original dataset for robust classification. It also aids in the visualization of data points during classification. The loadings matrix **P** provides information about the variables that make the largest contribution to the components.

Model development was achieved through five-fold cross-validation. The data were divided into five equal chunks; four chunks were used as the training set, and one was used as the validation set. This process was repeated by alternating each of the data chunks as a validation set and the remaining four as the training set. In each case, preprocessed data were loaded in the Matlab 2018 software workspace, and Matlab’s classification Learner apps were used to create the models. The pre-processed data consisted of 297 rows (representing 297 mosquitoes) and 341 features (wavenumbers). Training the classification models with all the features resulted into either underfitting for the linear models (LDA and LR) or a failed training for QDA. This necessitated feature extraction process by PCA. The Quadratic SVM (QSVM) model’s performance was largely unaffected by training with all the features. The PCA function in the software was used to automatically compress the input variables from 341 to 21 principal components, which accounted for 95% of the variation in the original datasets. The goal of feature extraction by PCA was to ensure robustness of the machine learning models. After the cross-validation process, the models were presented with completely new data from 100 mosquitoes (50 from each of the categories).

### Quality metrics for performance of the models

**Fig. 2:**
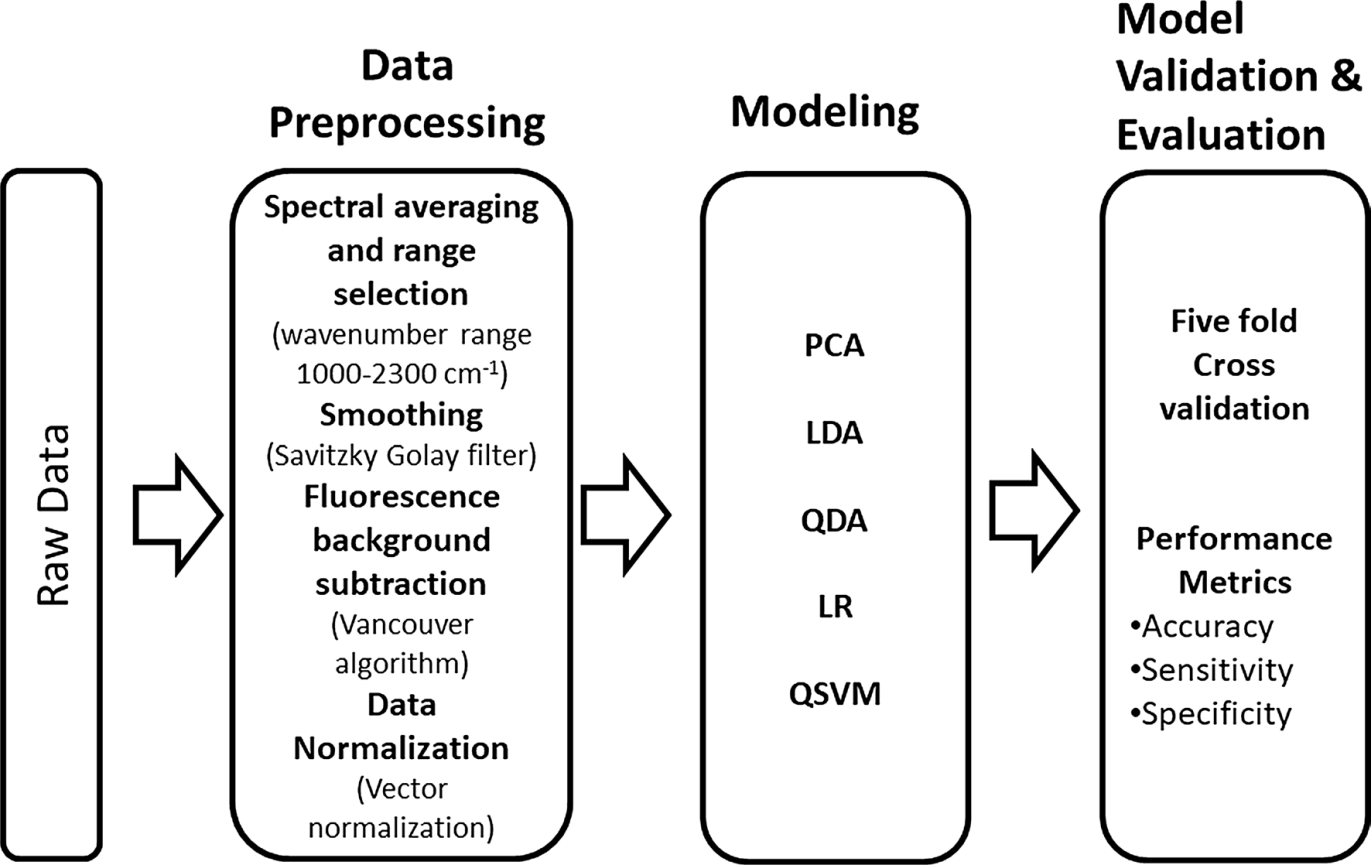
Data analysis pipeline. Raw data are fed from the left side of the figure. Pre-processing is performed before the clean data are fed into the classification models. Finally, the performances of the models are evaluated

Three quality metrics were calculated from the confusion matrices of the developed LDA, QDA, LR, and QSVM models to evaluate their performance: accuracy, sensitivity, and specificity. Accuracy is defined as the percentage of correct classifications, sensitivity is the percentage of true positives that were classified correctly, and specificity is the percentage of true negatives that received the correct classification. The metrics were calculated using Eqs. 2–4 [37].

2 $$Accuracy=\frac{TP+TN}{TP+FP+TN+FN}\times 100\%$$

3 $$Sensitivity=\frac{TP}{TP+FN}\times 100\%$$

4 $$ Specificity\, = \,\frac{TN}{{TN + FP}}\, \times \,100\% $$

where TP, TN, FP, and FN represent the True Positives, True Negatives, False Positives, and False Negatives, respectively. Figure 2 summarizes the data analysis protocol used in the development of the models.

## Results

**Fig. 3:**
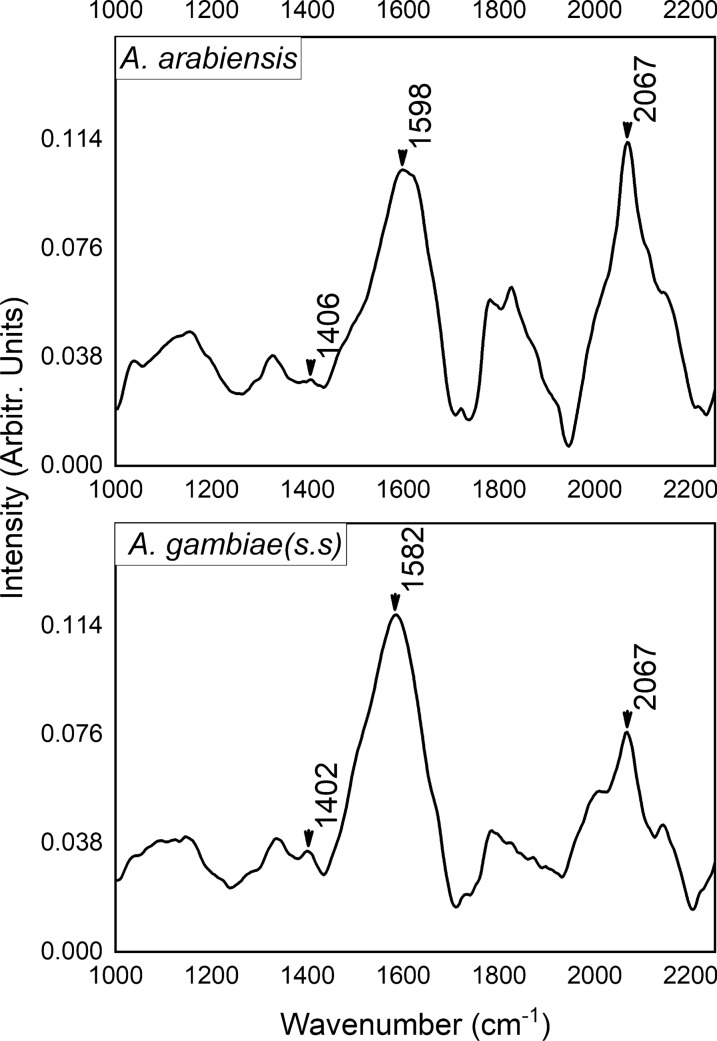
Average Raman spectra of *Anopheles arabiensis* (top) and *Anopheles gambiae* (bottom). The spectra are dominated by the eumelanin and pheomelanin peaks

The average Raman spectra obtained from the legs of the *An. gambiae* and *An. arabiensis* mosquitoes are shown in Fig. 3. Each spectrum is the average of the mean spectra from 20 individual mosquitoes. The main peaks of interest are labelled. These peaks coincide well with previously reported data on mosquito discrimination and classification [35]. Previous studies have shown that performing Raman spectroscopy on cuticles of some insects such as bumble bees [39], wasps [40], spiders [41] and skins of Lacertids [42] produced spectra dominated by peaks attributable to melanin pigment. The main peaks, as surveyed from the published work, occur at 1380 cm^−1^ and 1580 cm^−1^ for black or brown colour producing eumelanin and 1490 cm^−1^ and 2050 cm^−1^ for reddish or yellowish colour producing pheomelanin. The peak at 2067 cm^−1^ in Fig. 3 occurs in both *An. gambiae* and *An. arabiensis* spectra, but with clearly visible differences in intensity and is attributed to pheomelanin pigment. This corresponds to the 2050 cm^−1^ peak reported in the cited literature. This peak has been observed in Raman spectra of synthetic pheomelanin [43] as well as in spectra of pheomelanin from grasshoppers [44]. Previous studies have assigned it to overtones or combination bands. The overtone could be the first harmonic of the C-N band of pheomelanin reported by Galván et al*.* [45] to occur at 1150 cm^−1^ but spanned a range of wavenumbers 1141 cm^−1^, 1147 cm^−1^, and 1159 cm^−1^ [45]. This peak, although not well defined, can be observed as a hump at approximately 1100 cm^−1^ in Fig. 3. The peaks at 1402 cm^−1^ and 1582 cm^−1^ in *An. gambiae* and 1406 cm^−1^ and 1598 cm^−1^ in *An. arabiensis* spectra can be ascribed to eumelanin. These peaks have been associated with the stretching vibration of the hexagonal carbon rings in the molecular structure, the vibration of the six C–C bonds within the rings, and the vibration of C-H of methyl and methylene groups in the eumelanin polymers [46]. Although there was a clear difference in the peak position and intensity in the averaged spectra of the two species, there were considerable perceived similarities when examining individual mean spectra from each of the mosquitoes in the study. Therefore, the aforementioned differences cannot be used to predict mosquito species; hence, the need for machine learning classification models.

### Classification Models

All the four machine-learning models performed relatively well in discriminating and classifying the two sibling species during cross-validation. The linear models, LDA and LR, performed almost identically, with accuracies of 86% and 85%, respectively. The quadratic models QDA and QSVM achieved slightly higher accuracies of 89% and 93%, respectively. Table 1 summarizes the quality metrics of the four classification models. In all four models, *An. gambiae* and *An. arabiensis* were assigned positive and negative class labels, respectively.

**Table 1:** Summary of quality metrics used to assess the performance of the four classification models

Model	Accuracy(%)		Sensitivity (%)		Specificity (%)	
	Cross-validation set	Test set	Cross-validation set	Test set	Cross-validation set	Test set
Linear Discriminant Analysis (LDA)	86	79	82	68	90	90
Quadratic Discriminant Analysis (QDA)	89	81	84	66	93	96
Logistic Regression (LR)	85	82	84	74	87	90
Quadratic Support Vector Machine (QSVM)	93	93	90	90	95	96

Total number of mosquitoes used for cross-validation = 297 (*Anopheles gambiae*, n = 151; *Anopheles arabiensis*, n = 146); Total number of mosquitoes used for model testing = 100 (*Anopheles gambiae*, n = 50; *Anopheles arabiensis*, n = 50)

**Fig. 4:**
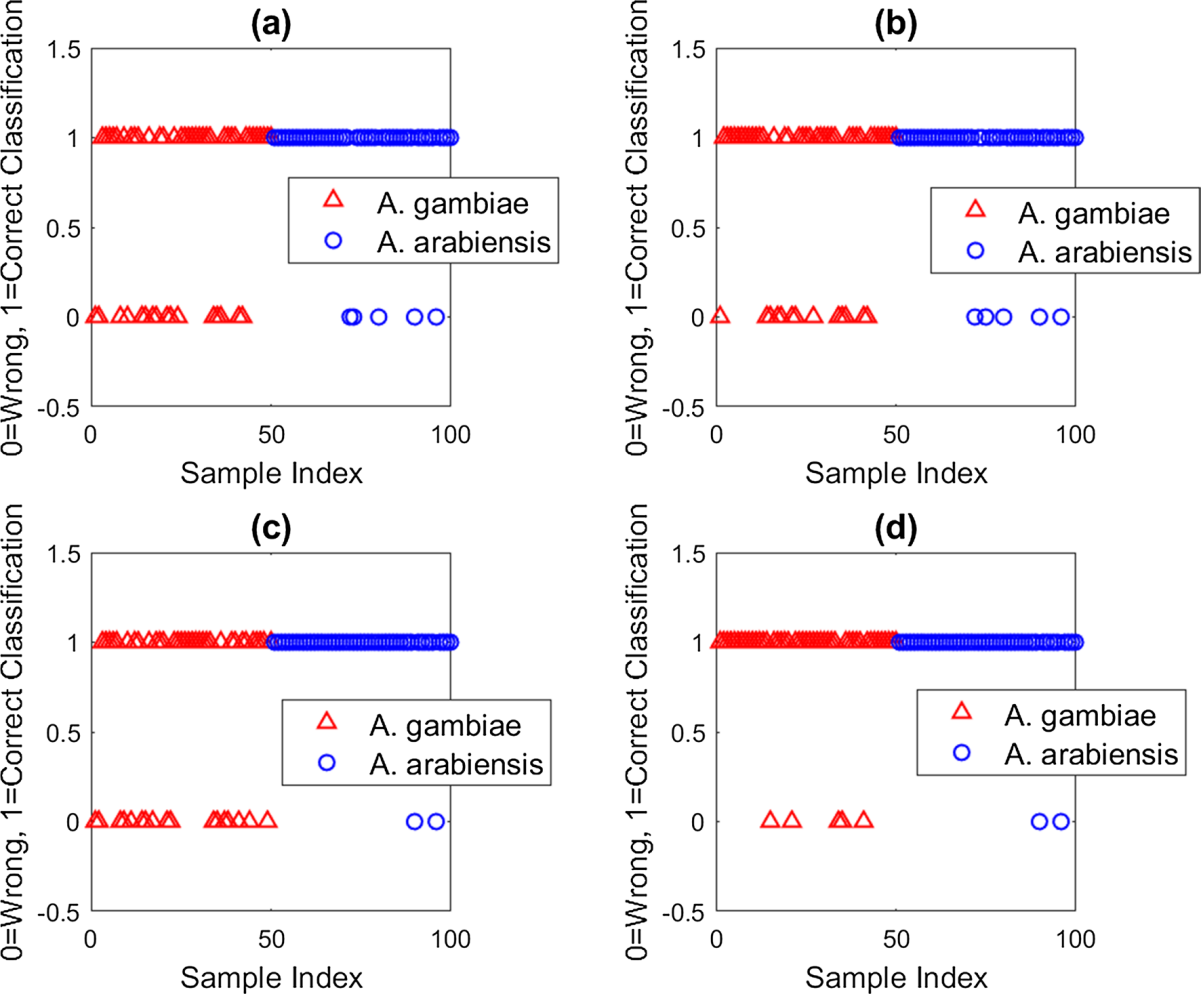
Model performance showing correct and wrong classification when presented with new unseen spectra: **a** LDA, **b** LR, **c** QDA and **d** QSVM. The data points represent each of the 100 mosquitoes (*Anopheles gambiae*, n = 50; *Anopheles arabiensis*, n = 50)

The overall prediction accuracies of the models were determined by exposing the models to new spectra from 100 mosquitoes (*An. gambiae*, n = 50; *An. arabiensis*, n = 50) that had not been seen by the models. LDA, LR, QDA and QSVM predicted correctly the two classes with accuracies of 79%, 82%, 81% and 93% respectively as summarized in Table 1. Figure 4 shows the model outputs for each of the new unseen samples.

### Variables contributions

**Fig. 5:**
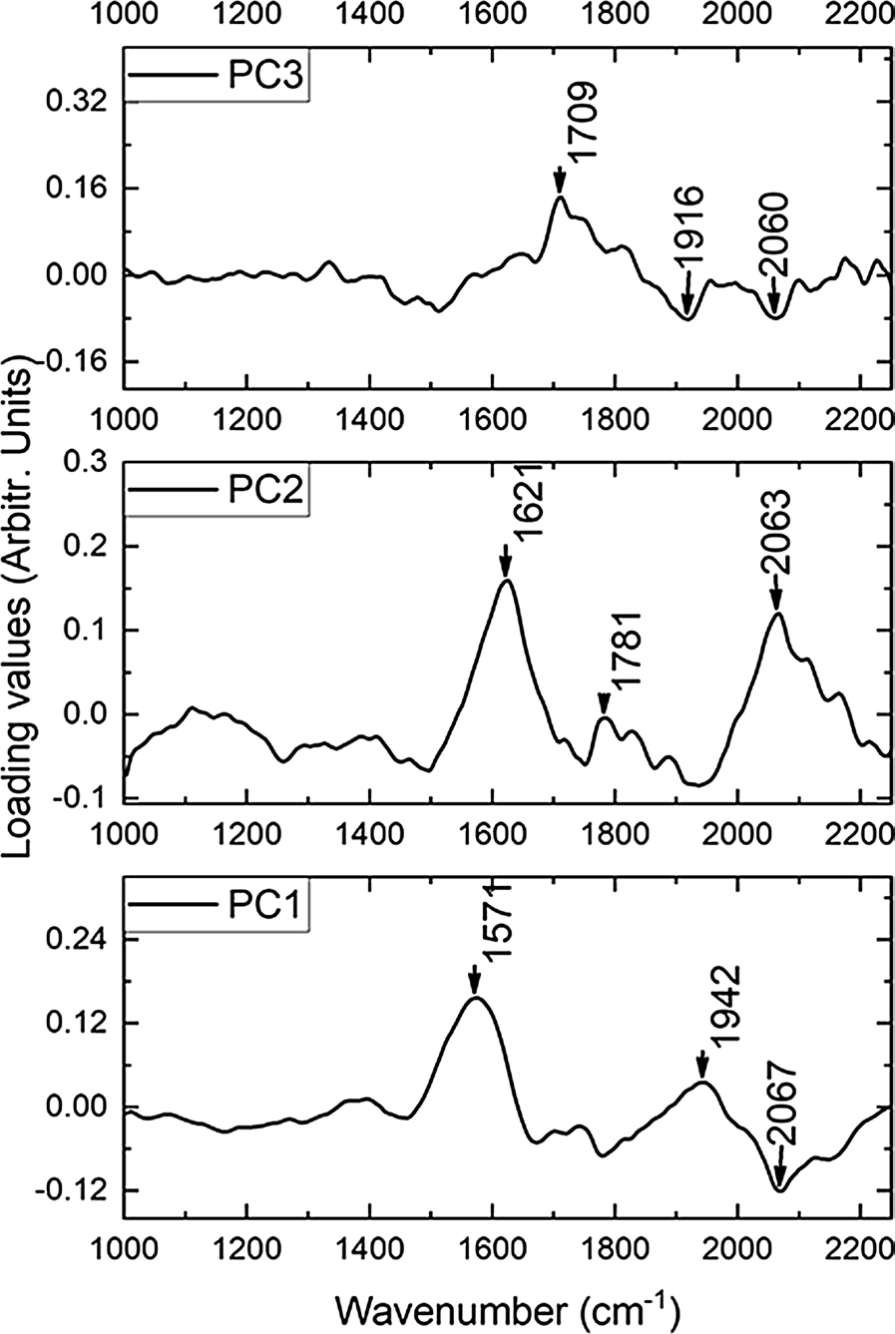
Loading visualization of the first three principal components. The loadings correlate with the Raman peaks of melanin. PC1, PC2, and PC3 accounted for 62.8%, 9.3%, and 4.2% of the variance, respectively

Each of the four models retained only twenty-one principal components (PCs) after training, accounting for 95.0% of the variation in the original feature space. The first three PCs accounted for 62.8%, 9.3%, and 4.2%, respectively, with each of the remaining 18 PCs contributing less than 3.0% of the variation. Examination of the loadings on these PCs revealed that the first PC correlated positively with the ca.1580 cm^−1^ eumelanin peak and negatively with the 2067 cm^−1^ pheomelanin peak. The second PC had strong positive loadings at 1621 cm^−1^ and 2063 cm^−1^. The third PC also showed a strong negative loading for pheomelanin characteristic bands at 2060 cm^−1^ and 1916 cm^−1^, and positive loading at 1709 cm^−1^, which are generally associated with carbonyl C = O bonds. Figure 5 provides a visualization of the loading for the first three PCs. Therefore, melanin peaks dominated in correlation with the first three PCs.

**Fig. 6:**
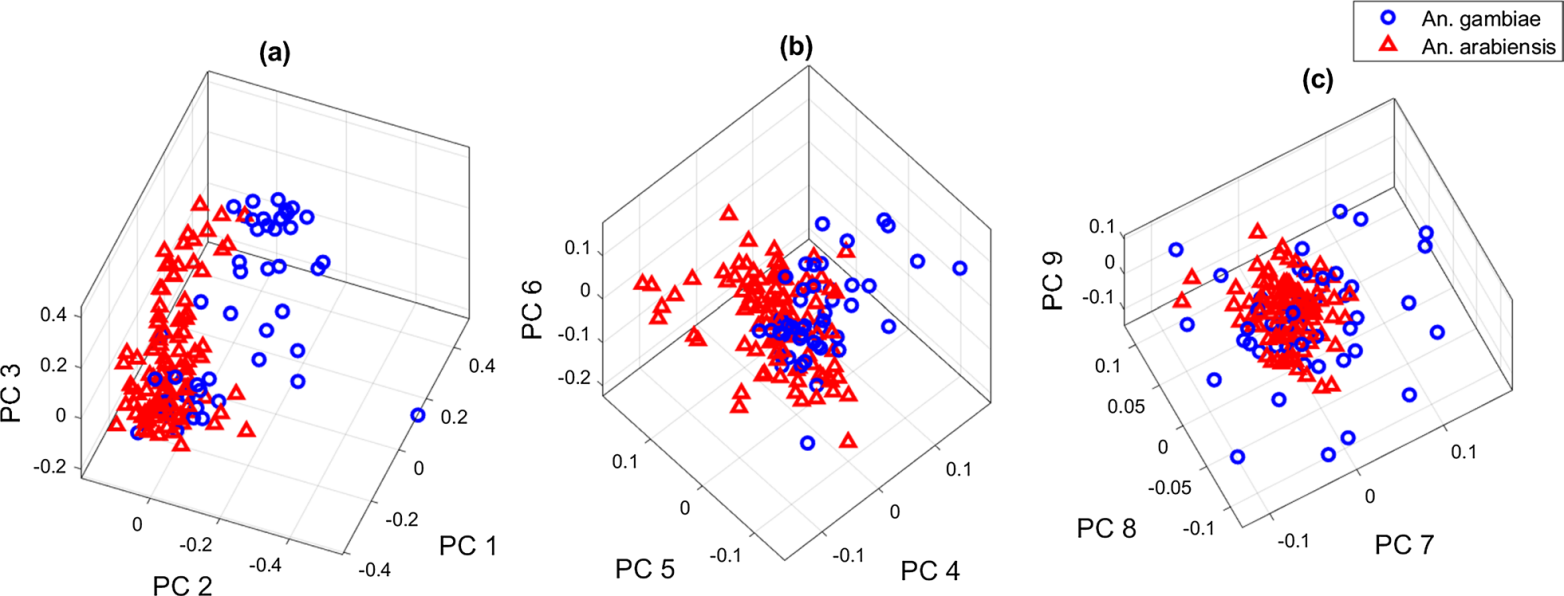
Visualization of principal component contribution in mosquito discrimination. Blue circles represent the positive group, whereas red triangles represent the negative group. The 3-D plots are **a** PC1,PC2, PC3, **b** PC4, PC5, PC6, and **c** PC7,PC8, PC9

While the classifiers used twenty-one PCs, it was not possible to visualize all PC combinations. Figure 6 shows a visualization of the mosquito data points in the principal component space in three dimensions. In Figs. 6a and b, there is a good separation between the positive (*An. gambiae*) and negative (*An. arabiensis*) classes using PC1, PC2, PC3 and PC4, PC5, PC6, respectively. In Fig. 6c, the positive class appears to be spread out more than the negative class, forming a smaller cluster in PC7, PC8, and PC9 space. This may be interpreted as small but important contributions by smaller PCs, which are useful for defining discrimination hyperplanes in the models.

## Discussion

The ability of Raman spectroscopy to effectively discriminate and classify two mosquito member species of the *An. gambiae* complex: *An. gambiae *sensu stricto and *An. Arabiensis* has been demonstrated. Both mosquito species were reared under controlled environmental conditions (humidity, temperature, and dark/light photoperiod). Raman spectra were obtained by scanning the cuticle of tibiae and femurs of unfed individual mosquitoes. After pre-processing, the spectra clearly showed the dominance of both eumelanin and pheomelanin peaks. Melanin molecules are known to offer photoprotective properties to organisms because of their ability to absorb ultraviolet (UV) and visible light and dissipate up to 90% of the absorbed energy. The thermal melanism hypothesis states that individuals tend to be darker in colder environments and lighter in warmer ones [47]. Since the measurements in this experiment were performed on individuals reared under similar environmental conditions, environment-induced changes in melanin were ruled out. Four machine-learning models, LDA, QDA, LR, and QSVM, were developed and successfully discriminated and classified the two cryptic species. They achieved accuracies of 86, 89, 85, and 93%, respectively on cross-validation. However, LDA, LR and QDA models showed some deterioration in performance when presented with completely new data set not used in cross-validation. This may be attributed to concept drift arising from measurements done at different dates with imprecise room temperature control. The general increase of specificity in the three models’ performances and a decrease in their sensitivity (Table 1) may validate this speculation. QSVM was largely more robust and unaffected by the drift. LDA and LR are linear models, whereas QDA and QSVM employ quadratic boundaries or kernels, respectively, in the discrimination algorithms. This suggests non-linearity in the datasets, and hence, better classification performance is exhibited by non-linear models. However, such models should be deployed with caution, such as cross-validation with sufficient data to avoid overfitting. The performance of the developed models can be considered sufficient for effective screening of large numbers of mosquito samples usually collected in mosquito surveillance programs and field studies.

The binary classification models LDA, QDA, LR, and QSVM developed in this work classify any sample presented to them into any of the two classes: *An. gambiae* and *An. arabiensis.* This means that before one can effectively use the models, one needs to sort the mosquitoes to the species level and then let the model assist in classifying the members of the *An. gambiae* complex. This is not different from the PCR assay, in which careful selection of primers and use of positive and negative controls are required to guarantee good results. However, unlike in the PCR assay where a failed amplification means the non-existence of the target species, a machine learning model classifies any sample presented to the model even if it does not belong to the complex to one of the classes, based on the learned decision boundary. This is a major weakness of the classification models, but it can be overcome by proper sorting of the insects and expanding the scope of the models to multiclass classifiers by training using all seven members of the complex. This may also include other members outside the complex, such as *Anopheles funestus*, which are occasionally confused with *An.gambiae* [48].

Compared with standard PCR assays, the method developed in this work is rapid because it requires minimal sample preparation. A fresh insect is placed under a microscope objective lens, as in normal microscopy, and scanned using a laser to acquire the Raman spectrum. For a case in which the model has been trained, the process of acquiring, preprocessing, and classifying the acquired spectrum can take less than ten minutes per insect. This time can be further reduced if a dedicated ‘point and shoot’ miniaturized system is developed and used. This can be considered faster than PCR, in which a sample can take between one and six hours depending on the speed of the system. It is also nondestructive, as the samples can be reused. After the extraction procedure for the PCR assay, an insect sample cannot be used for assays other than PCR. In terms of costs, after the initial costs of setting up the Raman microscope are considered, this method is relatively cost-saving because no chemical reagents are required. The Raman microscope used in this study costs approximately USD 100,000. However, it is also a general-purpose system that is used in other research projects in materials science, forensics, and bio-photonics. A point to note with Raman spectroscopy is that after the development of the method presented in this paper, a custom-made, application-specific, handheld system [49, 50] can be designed with preloaded libraries and search algorithms that may incorporate machine learning for mosquito identification. This will drastically decrease the initial cost of setting up a Raman system dedicated to mosquito identification to less than USD 30,000, and present a simplified user interface. The current cost of setting up a PCR system is approximately USD 40,000, with an expected constant requirement for reagents that may not be sustainable for laboratories in resource-limited settings. Finally, the Raman spectroscopy method also compares well with other optical spectroscopy techniques, MIR [32–34] and NIR [31, 51, 52] in mosquito identification studies. However, the use of ATR in MIR (ATR-MIR) spectroscopy on mosquitoes may potentially be considered destructive since the dried samples have to be pressed by an anvil onto a crystal during measurement. The ATR-MIR method also requires the samples to be dried for at least a day before acquisition of spectra, which is a source of delay. Raman spectroscopy overcomes such ATR-MIR challenges because measurement is done on fresh samples and without any sample damage. NIR spectroscopy, on the other hand, offers similar benefits to Raman spectroscopy (rapid and non-destructive) but from a technical perspective, Raman measurements are performed using laser light in (or close to) the visible range of the electromagnetic spectrum. Therefore, Raman spectroscopy is more appealing for the miniaturization of spectroscopic devices because visible light detectors and optics are relatively cheaper than those in NIR spectroscopy. Miniaturized Raman systems such as hand-held can be used to do measurements in situ while in field campaigns.

## Conclusions

The efficacy of a rapid and non-destructive method for discriminating and classifying two cryptic mosquito species belonging to the *An. gambiae* complex has been demonstrated. This method uses Raman spectroscopy combined with machine learning algorithms to discriminate between *An. gambiae* and *An. arabiensis*.

These results suggest that the cuticular pigment, melanin, is a biomarker for discriminating between the two sibling species. Linear binary models, namely Linear Discriminant Analysis (LDA) and Logistic Regression (LR), performed well with accuracies of 86% and 85%, respectively on cross-validation and 79%, 82%, respectively, on the test data set. Quadratic models -Quadratic Disciminant Analysis (QDA) and Quadratic Support Vector Machine QSVM- performed better than linear models, achieving accuracies of 89% and 93% respectively on cross-validation. QDA achieved 82% while QSVM remained robust achieving 93% accuracy respectively on the test data set. This is the first time Raman spectroscopy has been used to discriminate and classify cryptic mosquito species.

The classification results are based on measurements performed on mosquitoes reared under controlled ecological conditions. Therefore, environment-induced changes in the samples were ruled out. However, such variations should carefully be considered if the models were to be extended to field-caught mosquitoes.

Although the classification models presented in this work are binary, they can be upgraded by training using multi-class data to enable screening of all seven *An. gambiae* siblings and other species commonly confused with *An. gambiae*.

## Data Availability

The spectroscopy data used in this work can be obtained by sending an email request to the corresponding author.
